# Construction of a cuproptosis‐related lncRNA signature for predicting prognosis and immune landscape in osteosarcoma patients

**DOI:** 10.1002/cam4.5214

**Published:** 2022-09-21

**Authors:** Shumin Ni, Jinjiong Hong, Weilong Li, Meng Ye, Jinyun Li

**Affiliations:** ^1^ Department of Oncology and Hematology The Affiliated Hospital of Medical School of Ningbo University Ningbo China; ^2^ Department of Hand Surgery, Department of Plastic Reconstructive Surgery Ningbo No. 6 Hospital Ningbo China; ^3^ Department of Orthopedic Surgery Beilun District People's Hospital Ningbo China

**Keywords:** cuproptosis, immunotherapy, long non‐coding RNA, osteosarcoma, prognosis

## Abstract

**Background:**

Long noncoding RNAs (lncRNAs) influence the onset of osteosarcoma. Cuproptosis is a novel cell death mechanism. We attempted to identify a cuproptosis‐related lncRNA signature to predict the prognosis and immune landscape in osteosarcoma patients.

**Methods:**

Transcriptional and clinical data of 85 osteosarcoma patients were derived from the TARGET database and randomly categorized into the training and validation cohorts. We implemented the univariate and multivariate Cox regression, along with LASSO regression analyses for developing a cuproptosis‐related lncRNA risk model. Kaplan–Meier curves, C‐index, ROC curves, univariate and multivariate Cox regression, and nomogram were used to assess the capacity of this risk model to predict the osteosarcoma prognosis. Gene ontology, KEGG, and Gene Set Enrichment (GSEA) analyses were conducted for determining the potential functional differences existing between the high‐risk and low‐risk patients. We further conducted the ESTIMATE, single‐smaple GSEA, and CIBERSORT analyses for identifying the different immune microenvironments and immune cells infiltrating both the risk groups.

**Results:**

We screened out four cuproptosis‐related lncRNAs (AL033384.2, AL031775.1, AC110995.1, and LINC00565) to construct the risk model in the training cohort. This risk model displayed a good performance to predict the overall survival of osteosarcoma patients, which was confirmed by using the validation and the entire cohort. Further analyses showed that the low‐risk patients have more immune activation and immune cells infiltrating as well as a good response to immunotherapy.

**Conclusions:**

We developed a novel cuproptosis‐related lncRNA signature with high reliability and accuracy for predicting outcome and immunotherapy response in osteosarcoma patients, which provides new insights into the personalized treatment of osteosarcoma.

## Graphical abstract

Using the TARGET‐OS database, we developed a risk score prognosis model using the cuproptosis‐related lncRNAs. The novel cuproptosis‐related lncRNA signature have high reliability and accuracy for predicting outcome and immunotherapy response in osteosarcoma patients.

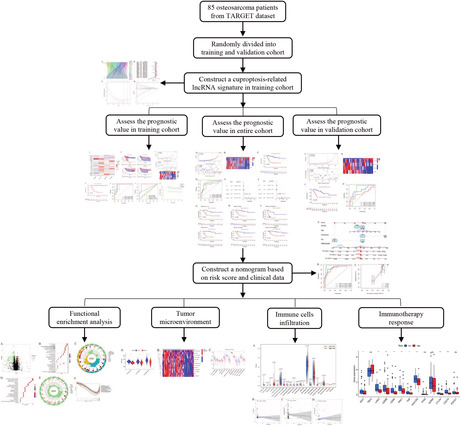

## 1 INTRODUCTION

Osteosarcoma is a very prevalent form of malignant bone cancer that is primarily detected in teenagers and young adults.^1^, ^2^ The primary affecting site of this tumor is generally noted in the metaphysis of the long bones, like the proximal tibia, distal femur, and proximal humeral.^3^ Osteosarcoma shows poor prognosis owing to its higher rate of recurrence and distant metastasis, particularly lung metastasis.^4^, ^5^ Over the past 50 years, although many advanced treatment strategies have been used for osteosarcoma, such as chemotherapy, limb‐sparing surgery, amputation, stereotactic radiotherapy, and immunotherapy, there has not been a significant improvement in the survival rate of the osteosarcoma patients, and the 5‐year survival value is approximately 60–70%.^6^, ^7^ Owing to the lack of accurate and reliable biomarkers, approximately 20% of all osteosarcoma patients show the presence of metastases upon diagnosis, which can challenge the management of osteosarcoma patients.^8^ Additionally, due to genetic heterogeneity, those osteosarcoma patients with the same treatments might have different prognoses.^9^, ^10^ Therefore, the molecular mechanisms underlying osteosarcoma metastasis and progression should be further explored. Besides, more reliable and efficient prognostic biomarkers are urgently identified to design novel techniques for treating and determining the prognosis of osteosarcoma patients.

Long noncoding RNAs (LncRNAs) are a subtype of noncoding RNAs and have >200 nucleotides.^11^ In the past few years, many researchers have presented evidence proving that the lncRNAs show a wide range of biological functions in regulating diverse physiological and pathological progression,^12^ including chromatin remodeling,^13^ transcriptional regulation, and posttranslational modification.^14^ Numerous studies have observed that the mutations and dysregulations in the lncRNAs cause many human diseases, such as cerebrovascular diseases,^15^ ischemic injuries,^16^ endocrinologies,^17^ immune system diseases,^18^ and especially cancer.^19^, ^20^, ^21^ Due to the characteristics of tissue specificity, high stability, abundance, and species conservation, lncRNAs have been identified as a possible diagnostic and prognostic biomarker in the clinical management of cancer.^22^ Additionally, lncRNAs are also associated with tumor metabolism, tumor microenvironment, and drug resistance,^23^ consequently recognized as potential cancer drug targets.^24^, ^25^

Programmed cell death is an essential physiological and pathological process to remove superfluous or damaged cells to ensure healthy development and tissue homeostasis,^26^ including apoptosis, autophagy, pyroptosis, ferroptosis, and necroptosis.^27^, ^28^, ^29^ Recently, a novel cell death pathway was reported by Tsvetkov et al.^30^ Cuproptosis is defined as the copper‐dependent death of the cells, where the copper binds directly to the lipoylated components of the tricarboxylic acid (TCA) cycle. It is well known that copper is an important cofactor that is essential for the activity of many cellular enzymes in all living organisms.^31^, ^32^ Excess copper causes mitochondrial protein aggregation and triggers cuproptosis.^33^ Several researchers have shown that the level of copper in cancer patients was higher than in healthy controls.^34^ Additionally, programmed cell death was seen to play a dual role in tumor regulation, as it stimulates tumor progression and also prevents tumor progression. Previous research showed that copper can induce tumor cell death as it helps in accumulating the reactive oxygen species (ROS), inhibits proteasomes, and causes antiangiogenesis.^35^ Therefore, these new findings invigorated studies exploring the use of copper to treat cancer. However, the mechanism of cuproptosis in osteosarcoma progression is still unclear, and the research regarding the role played by cuproptosis‐related lncRNAs in osteosarcoma is still not completely clear.

In this study, we screened the cuproptosis‐related lncRNAs in osteosarcoma patients from the TARGET database to develop a prognostic model and validate the capacity of this model for anticipating the prognosis of osteosarcoma patients. We also carried out the functional enrichment analysis using the data of the high‐risk and low‐risk patients for investigating the underlying mechanism of cuproptosis‐related lncRNAs in osteosarcoma. Finally, we examined the relationship between the novel risk model and immune characteristics present in osteosarcoma patients. These results could offer a lot of useful data that could improve the prognosis of osteosarcoma patients.

## 2 MATERIALS AND METHODS

### 2.1 Data source

We downloaded the transcriptome sequencing data and the clinical information related to the 88 osteosarcoma patients from the Therapeutically Applicable Research to Generate Effective Treatments (TARGET, https://ocg.cancer.gov/programs/target) database. After filtering out three samples without survival information, we randomly categorized the 85 patients into the training (*n*  =  43) and validation (*n*  =  42) cohorts, in a 1:1 ratio for further analysis. Table 1 presents the clinical data of all patients. Table 2 presents the clinical features of patients in the training and validation cohorts.

**TABLE 1:** Clinical features of all osteosarcoma patients in this study

Covariates	Type	Target	
		Number	Percent
Age	≤14	39	44.32%
	>14	45	51.14%
	Unknown	1	1.14%
Gender	Female	37	42.05%
	Male	47	53.41%
	Unknown	1	1.14%
Race	White	51	57.95%
	Asian	6	6.82%
	Black or African American	7	7.95%
	Unknown	21	23.86%
Primary tumor site	Leg	76	86.36%
	Arm	6	6.82%
	Pelvis	2	2.27%
	Unknown	1	1.14%
Metastasis status	Yes	21	23.86%
	No	63	71.59%
	Unknown	1	1.14%
Survival status	Dead	27	30.68%
	Alive	58	65.91%

**TABLE 2:** Clinical features of all osteosarcoma patients categorized into the training and validation cohorts

Covariates	Type	Total	Train	Test	*p* value
Age	≤14	39 (45.88%)	18 (41.86%)	21 (51.22%)	0.5216
	>14	45 (52.94%)	25 (58.14%)	20 (48.78%)	
	Unknown	1 (1.18%)	0	1 (2.38%)	
Gender	Female	37 (43.53%)	22 (51.16%)	15 (35.71%)	0.2604
	Male	47 (55.29%)	21 (48.84%)	26 (61.91%)	
	Unknown	1 (1.18%)	0	1 (2.38%)	
Metastasis	No	63 (74.12%)	34 (79.07%)	29 (69.05%)	0.5286
	Yes	21 (24.71%)	9 (20.93%)	12 (28.57%)	
	Unknown	1 (1.18%)	0	1 (2.38%)	
Primary tumor site	Arm	6 (7.06%)	3 (6.98%)	3 (7.14%)	0.9975
	Leg	76 (89.41%)	39 (90.7%)	37 (88.10%)	
	Pelvis	2 (2.35%)	1 (2.33%)	1 (2.38%)	
	Unknown	1 (1.18%)	0	1 (2.38%)	

### 2.2 Identification of the cuproptosis‐related lncRNAs

We acquired 19 cuproptosis‐related genes from the previously reported literature.^33^, ^35^, ^36^ We extracted the list of cuproptosis‐related genes and identified the cuproptosis‐related lncRNAs using the |correlation coefficient| > 0.4 and a *p*‐value <0.001, with the help of the “limma” package. Then, we conducted the univariate Cox regression analysis for screening and detecting the prognostic cuproptosis‐related lncRNAs after setting a threshold value of *p*‐value <0.05.

### 2.3 Development and validation of the cuproptosis‐related lncRNA prognostic signature

Using the training cohort, we carried out the Least Absolute Shrinkage and Selection Operator (LASSO) regression analysis for optimizing the cuproptosis‐related prognostic lncRNAs and preventing data overfitting. We also conducted the multivariate Cox analysis for simplifying the number and estimating the coefficient of cuproptosis‐related lncRNAs in the risk model. We determined the risk score of every patient included in the training and validation cohorts, using the formula below:

$$\begin{array}{c} \text{Risk score}=\sum_{i=1}^{n}\text{coefi}\times \text{cuproptosis}\\ -\text{related lncRNA expression} \end{array}$$

We categorized osteosarcoma patients into high‐ and low‐risk groups, based on the median risk score value of the training set. Then, we used the principal component analysis (PCA) for determining whether this signature could distinguish the osteosarcoma patients between the two groups. We used the Kaplan–Meier curve, univariate Cox regression, and multivariate Cox regression analyses for assessing the prognostic value of cuproptosis‐related lncRNA signature. We also used the receiver operator characteristic (ROC) curves along with the C‐index for assessing the model accuracy. To determine whether our cuproptosis‐related lncRNA had a superior predictive ability for osteosarcoma patients, we compared it with three published lncRNA prognostic signatures related to pyroptosis,^37^ iron metabolism,^38^ and N6‐methyladenosine.^39^

### 2.4 Construction of nomogram

We used the risk scores and the clinical factors to develop a nomogram that could accurately anticipate the 1‐, 3‐, and 5‐year overall survival (OS) rate of osteosarcoma patients. We used the time‐dependent calibration and ROC curves for evaluating the ability of the novel nomogram for prognosis prediction.

### 2.5 Functional enrichment analysis

We used the “limma” package for screening the differentially expressed genes (DEGs) between the high‐risk and low‐risk groups with |log2FC| >1 and FDR *q* < 0.05. We further carried out the GO (i.e., Molecular Function, Biological Processes, and Cellular Component) and KEGG enrichment analyses on these DEGs using the “clusterProfiler” package. Subsequently, we performed the Gene Set Enrichment Analysis (GSEA) between high‐risk and low‐risk groups.^40^

### 2.6 Correlation between the cuproptosis‐related lncRNAs prognostic signature and immune characteristics

ESTIMATE package^41^ was used to calculate the ImmuneScore, StromalScore, and ESTIMATEScore of osteosarcoma patients. The ESTIMATEScore was calculated as the sum of the ImmuneScore and the StromalScore and indicated the general ratio of these factors in the tumor microenvironment (TME). We conducted the single‐sample GSEA (ssGSEA)^42^ to determine the activity of the immune‐linked functions between the high‐ and low‐risk groups, applying the GSVA package. We used the CIBERSORT technique^43^ for analyzing the infiltration levels of the 22 immune cells in the patients categorized into both risk groups. We analyzed the correlation between the proportion of infiltrating immune cells and the risk scores. Last, we compared and assessed the expression of the immune checkpoint‐related genes (ICGs) among patients in both risk groups, with the help of the “reshape2,” “limma,” “ggplot2,” and “ggpubr” packages, for determining the response of the osteosarcoma patients to immunotherapy.

### 2.7 Statistical analysis

We used the R software (v4.1.0) to statistically analyze all data and visualize the results. We conducted the Chi‐square and the Wilcoxon signed‐rank tests to compare the difference observed across subgroups. We used the log‐rank test for comparing the Kaplan–Meier survival curves. We also conducted the univariate and multivariate Cox regression analyses for estimating the hazard ratio (HR) and the 95% confidence interval (CI) values for the risk scores and other important clinical parameters. We used the ROC curves and the values of the area under the curve (AUC) for determining the predictive accuracy of the risk model. A *p* value of <0.05 was considered statistically significant.

## 3 RESULTS

### 3.1 Identifying the cuproptosis‐related prognostic lncRNAs in the osteosarcoma patients

**FIGURE 1:**
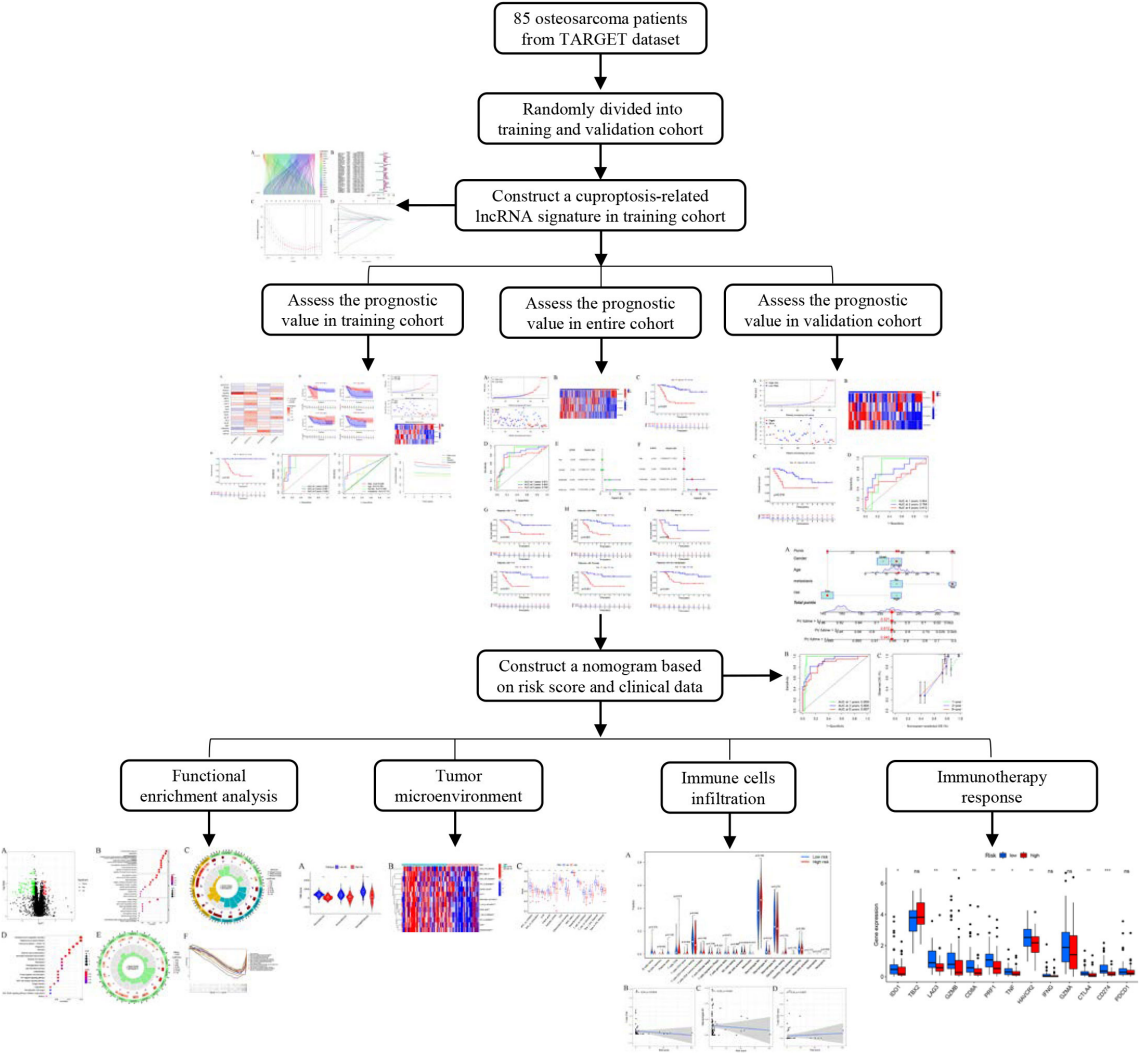
The flow chart of this study.

**FIGURE 2:**
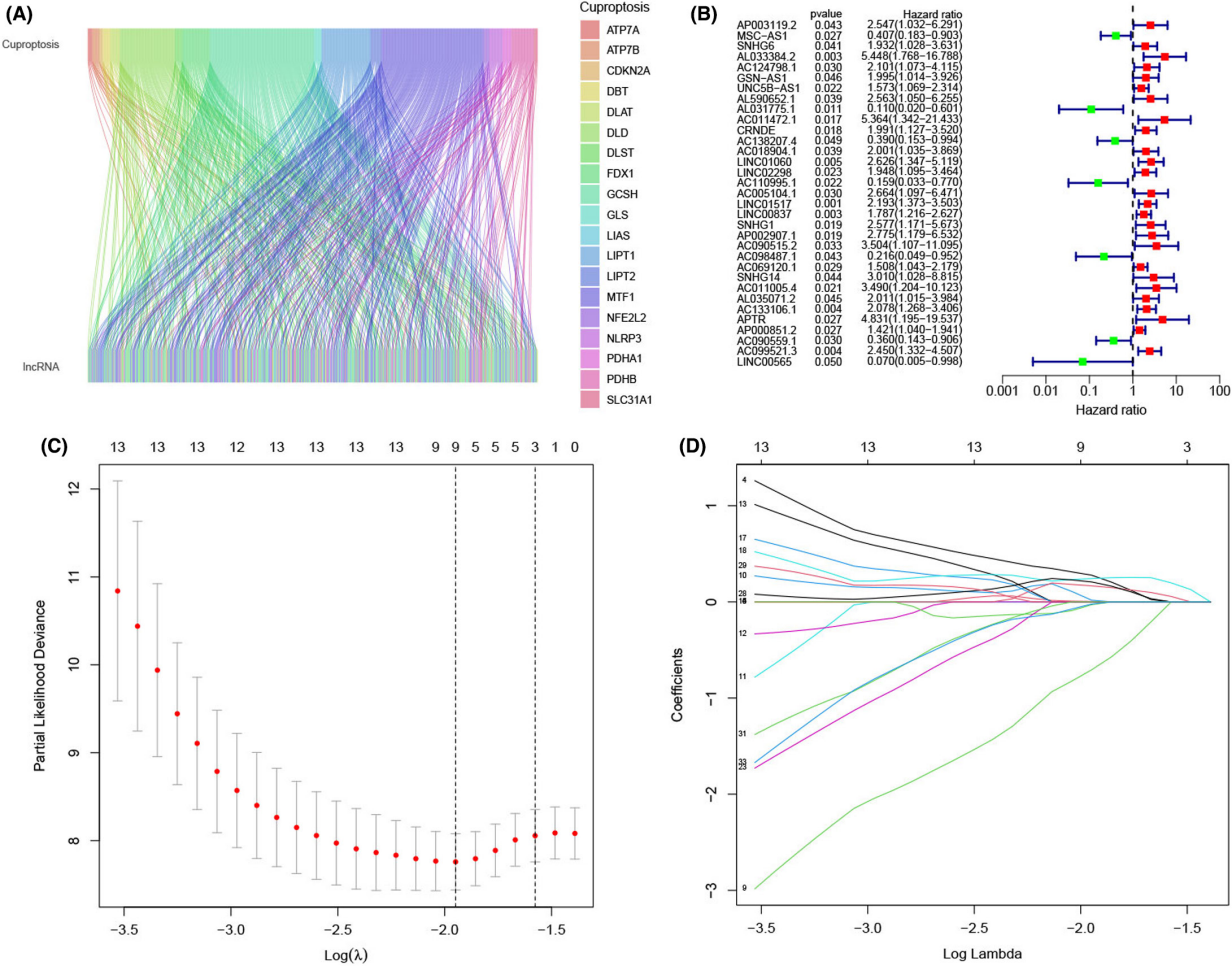
Developing a four cuproptosis‐related long noncoding RNA (lncRNAs) signature for osteosarcoma patients. (A) Sankey diagram highlighting the relationship between the cuproptosis genes and the cuproptosis‐related lncRNAs. (B) Forest plot highlighting the univariate Cox regression analysis results. (C) A general cross‐validation curve of the paired likelihood deviance. (D) Elucidation for the LASSO coefficient profiles of the prognostic lncRNAs.

The flow chart of this study is shown in Figure 1. First, we recorded the expression levels of 19 cuproptosis‐related genes derived from the TARGET‐OS cohort. Thereafter, we carried out the Pearson correlation analysis to identify 696 cuproptosis‐related lncRNAs (Table S1), wherein their expression levels could be related to a single or multiple cuproptosis‐related genes (Figure 2A). Last, we screened 33 of these lncRNAs using the univariate Cox regression analysis, which served as the cuproptosis‐related prognostic lncRNAs in osteosarcoma patients for further analysis (Figure 2B).

### 3.2 Developing a cuproptosis‐related lncRNA prognostic model

**FIGURE 3:**
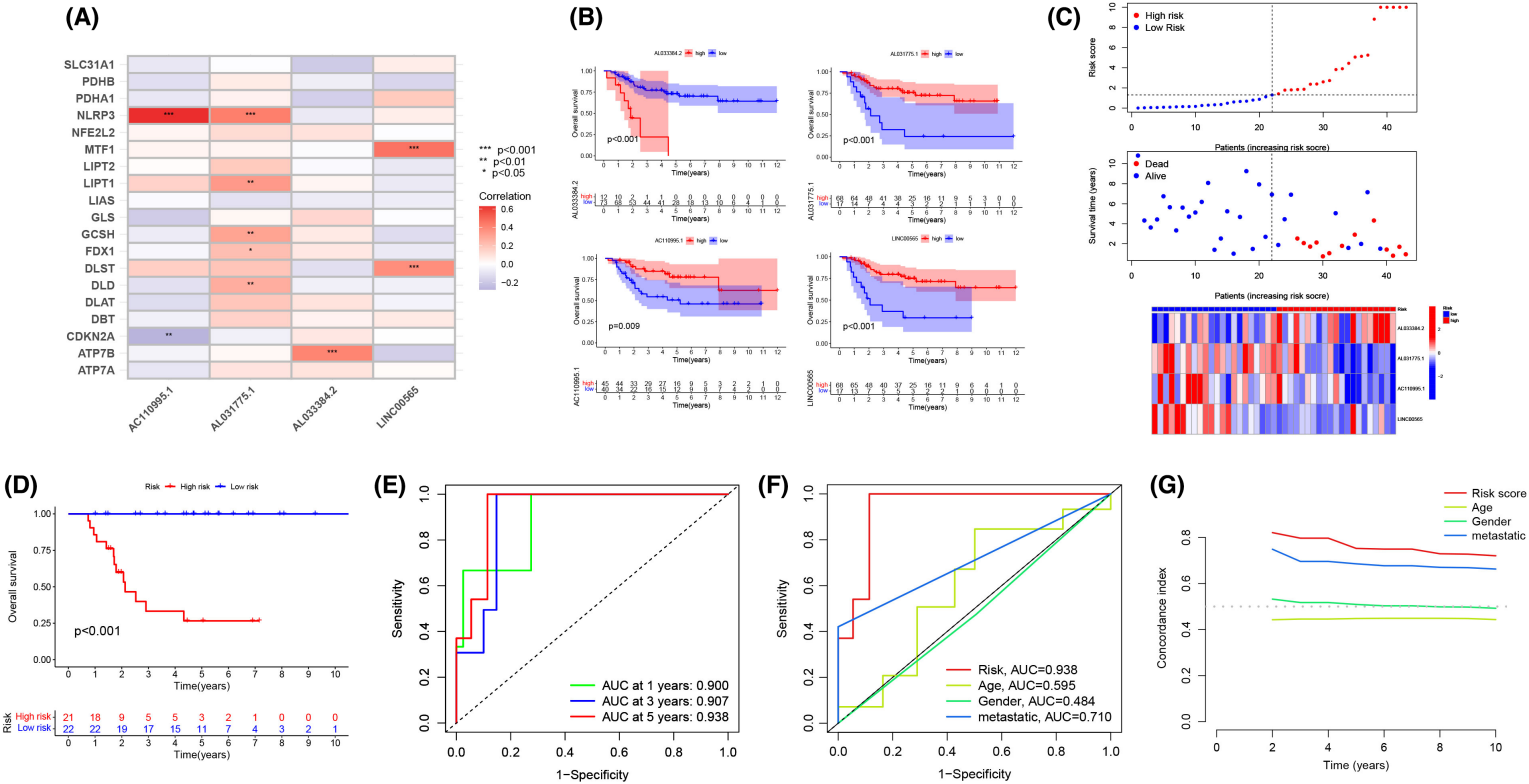
The prognosis of cuproptosis‐related lncRNA signature using the training cohort. (A) Relationship between the cuproptosis genes and four cuproptosis‐related lncRNAs in the training cohort. (B) Kaplan–Meier curves for the four lncRNAs in the signature. (C) Distribution of the overall survival (OS) state, OS time, and risk scores of the data included in the training cohort. (D) Kaplan–Meier curve for the OS of the high‐ and low‐risk patients included in the training cohort. (E) The area under the curves (AUCs) of the time‐dependent receiver operator characteristic (ROC) curves validated the prognostic accuracy of the risk scores in the training cohort. (F) AUCs of the ROC curves in comparison to the prognostic accuracy of risk scores and other clinical parameters in the training cohort. (G) The C‐index was used for determining the prognostic accuracy of risk scores and a few additional clinical factors in the training cohort.

To develop a cuproptosis‐related lncRNA prognostic model, we categorized 85 osteosarcoma patients into the training and validation cohorts (1:1 ratio), using the random sampling technique. Then, in the training cohort, we used the LASSO regression analysis and introduced a new lambda value in the data set to decrease the total number of variables (Figure 1C,D). We further used the multivariate Cox regression analysis for screening four lncRNAs out of the remaining nine lncRNAs and calculating the coefficients. Figure 3A presents the correlation between the four LncRNAs incorporated in the signature and cuproptosis‐related genes. The Kaplan–Meier analysis results (Figure 3B) indicated that the osteosarcoma patients who had a higher AL033384.2 expression level showed a worse prognosis. On the other hand, patients with a higher AL031775.1, AC110995.1, and LINC00565 expression level showed a favorable prognosis. We calculated the risk score as follows: Expression value of AL033384.2 × 1.743 + expression value of AL031775.1 × −1.495 + expression value of AC110995.1 × −1.785 + expression value of LINC00565 × −2.283.

### 3.3 Validating the established risk model

**FIGURE 4:**
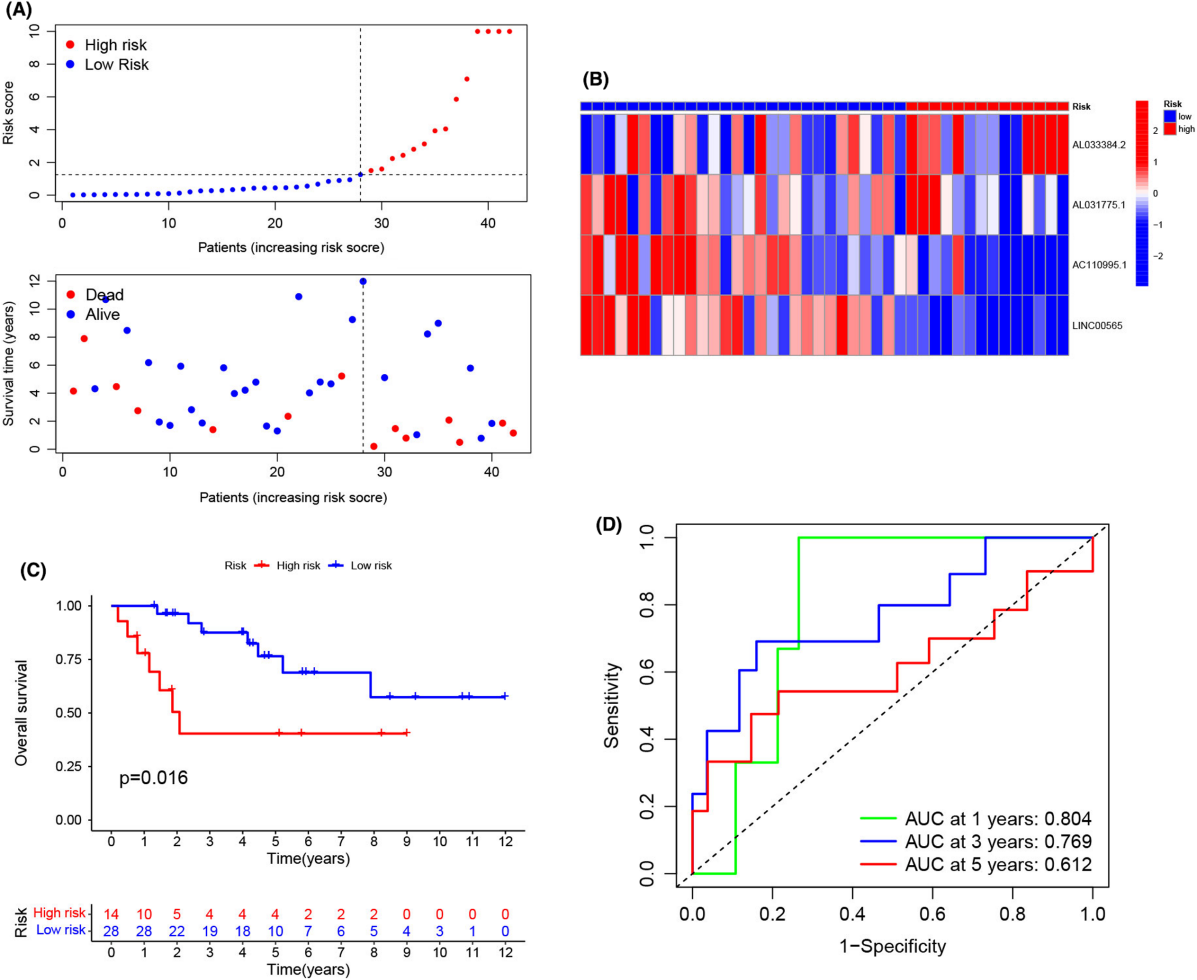
Prognosis of the cuproptosis‐related lncRNA signature in the validation cohort. (A) Distribution of the overall survival (OS) time, OS state, and risk scores for patients categorized into the validation cohort. (B) Heatmap of the four cuproptosis‐related lncRNA expression profiles in the validation cohort. (C) Kaplan–Meier curve for the OS of the high‐ and low‐risk patients included in a validation cohort. (D) AUCs of time‐dependent receiver operating characteristic curves validated the prognostic accuracy of the risk scores in the validation cohort.

**FIGURE 5:**
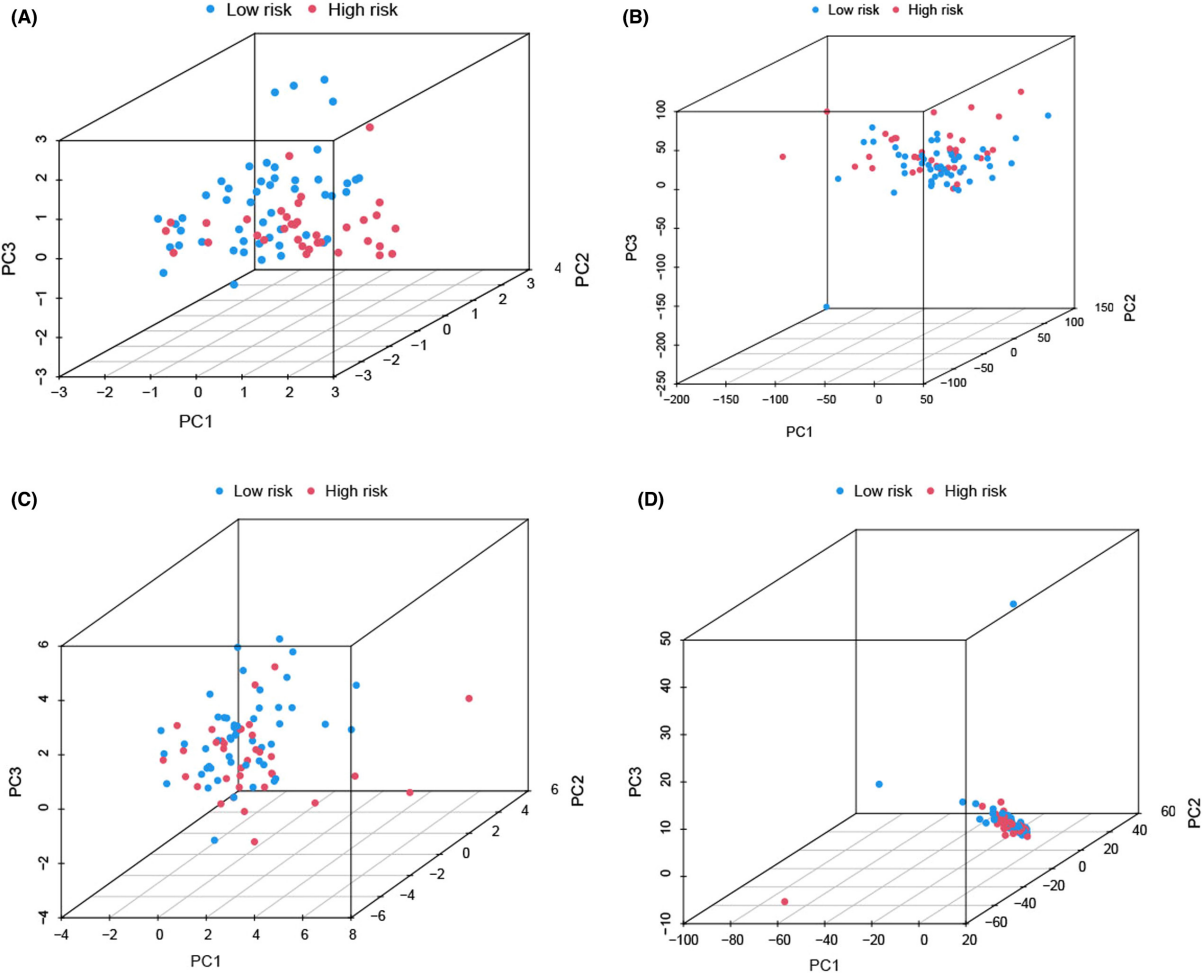
Principal component analysis. Four cuproptosis‐related lncRNA signatures (A) could better distinguish the risk status of patients than the whole‐genome expression (B), cuproptosis‐related genes (C), and cuproptosis‐related lncRNA (D).

**FIGURE 6:**
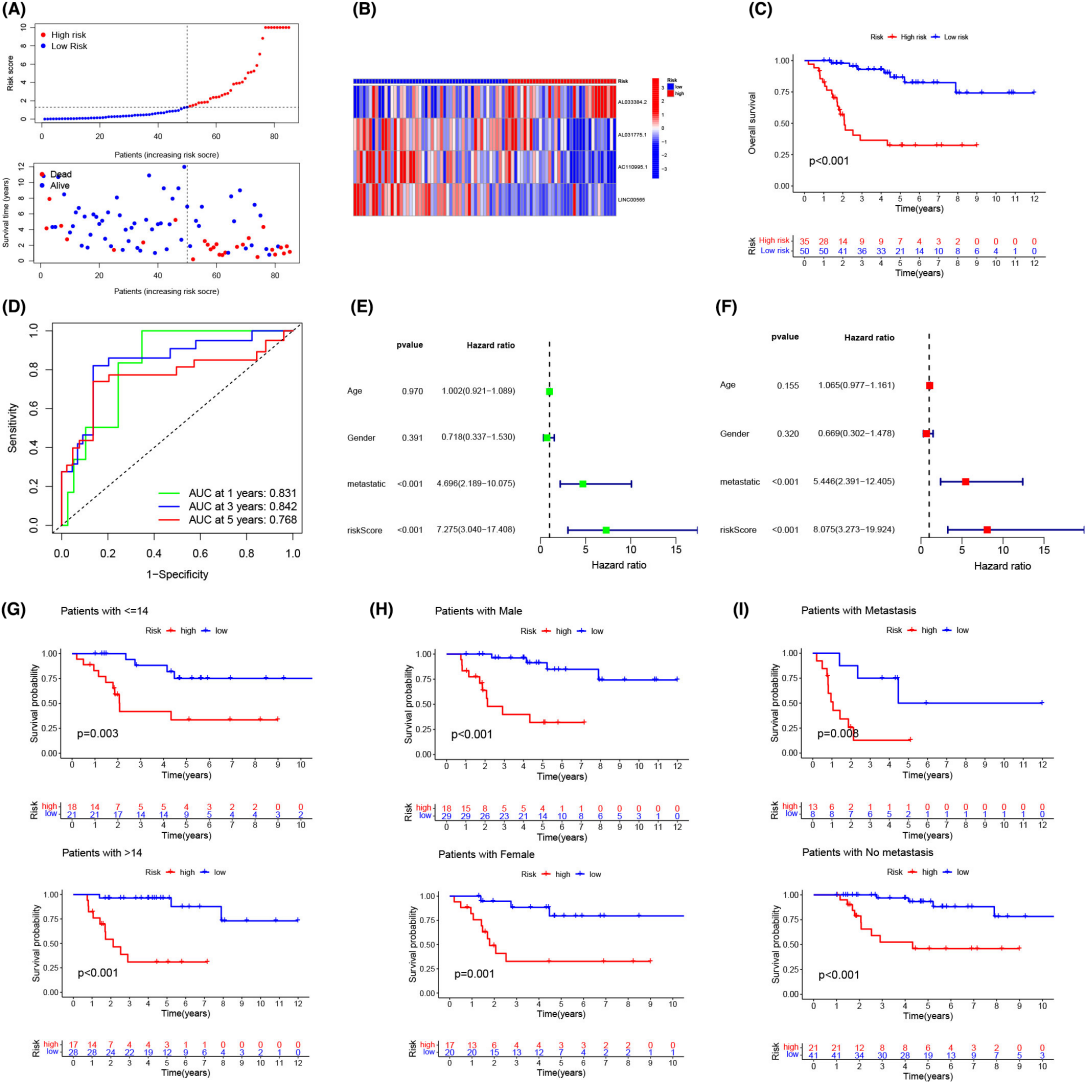
Prognosis of the cuproptosis‐related lncRNA signature in osteosarcoma patients. (A) Distribution of the overall survival (OS) time, OS state, and risk scores in all patients. (B) Heatmap of four cuproptosis‐related lncRNA expression profiles in all samples. (C) Kaplan–Meier curves for the OS of the high‐ and low‐risk osteosarcoma patients. (D) The area under the curves of the time‐dependent ROC curves that validated the prognostic accuracy of risk scores in all samples. (E) Univariate Cox regression analysis of cuproptosis‐related lncRNA signature in all samples. (F) Multivariate cox regression analysis of cuproptosis‐related lncRNA signature in all samples. (G) Subgroup analysis of the Kaplan–Meier curve related to the patients' age. (H) Subgroup analysis of the Kaplan–Meier curves related to the patients' gender. (I) Subgroup analysis of the Kaplan–Meier curves related to the patients' metastasis status.

After calculating the risk score values, we categorized the patients from the training and validation cohorts into the high‐ and low‐risk groups depending on the median value of the training cohort. Figure 3C presents the results of the risk score distribution, OS state of osteosarcoma patients, and expression levels of four LncRNAs in the training cohort. The Kaplan–Meier curve revealed that the high‐risk osteosarcoma patients showed a poor prognosis in comparison with the low‐risk patients in the training cohort (Figure 3D). The AUCs of ROC curves for the 1‐, 3‐, and 5‐year OS (Figure 3E) were 0.900, 0.907, and 0.938, respectively. However, the other clinical factors showed lower values (Figure 3F; age: 0.595; gender: 0.484; and metastasis: 0.710). Furthermore, as depicted in Figure 3G, this risk score showed the highest C‐index compared with other clinical factors. Figure 4A presents the distribution of the risk scores and the OS state of osteosarcoma patients included in the validation cohort. Figure 4B presents the expression levels of four LncRNAs in osteosarcoma patients categorized into the high‐ and low‐risk groups. Similar to the results noted for the training cohort, the Kaplan–Meier curve (Figure 4C) for the validation cohort revealed that the high‐risk patients displayed a poor OS than the low‐risk patients. The AUC values for ROC curves developed for the 1‐, 3‐, and 5‐year OS for patients in the validation cohort were seen to be 0.804, 0.769, and 0.612, respectively (Figure 4D). We then merged both the training and the validation cohorts for increasing the sample size. We carried out the PCA analysis of the entire cohort and noted that the signature incorporating four cuproptosis‐related lncRNAs (Figure 5A) could differentiate the risk status of the osteosarcoma patients more effectively in the whole genome expression (Figure 5B), cuproptosis‐related genes (Figure 5C), and the cuproptosis‐related lncRNA (Figure 5D). Figure 6A presents the risk score distribution and the OS state of osteosarcoma patients included in the entire cohort. Figure 6B describes the expression level of the four cuproptosis‐related LncRNAs in the high‐ and low‐risk patients. The Kaplan–Meier curve (Figure 6C) developed for the entire cohort demonstrated that the high‐risk patients showed a short OS compared to the low‐risk patients. For the entire cohort, the AUCs of ROC curves for the 1‐, 3‐, and 5‐year OS were 0.831, 0.842, and 0.768, respectively (Figure 6D), which were higher than that previous LncRNA signature for osteosarcoma patients (Table S1). Univariate (Figure 6E) and multivariate cox regression analyses (Figure 6F) indicated that the risk scores (HR  =  8.075, *p* < 0.001) and metastasis status (HR  =  5.446, *p* < 0.001) can be regarded as independent prognostic parameters for osteosarcoma patients. The OS analysis of the subgroups depending on factors like age (Figure 6G), gender (Figure 6H), and metastatic state (Figure 6I) revealed that the high‐risk patients displayed a poor prognosis compared to the low‐risk patients in every subgroup.

### 3.4 Development of a novel nomogram based on the risk scores and clinical data

**FIGURE 7:**
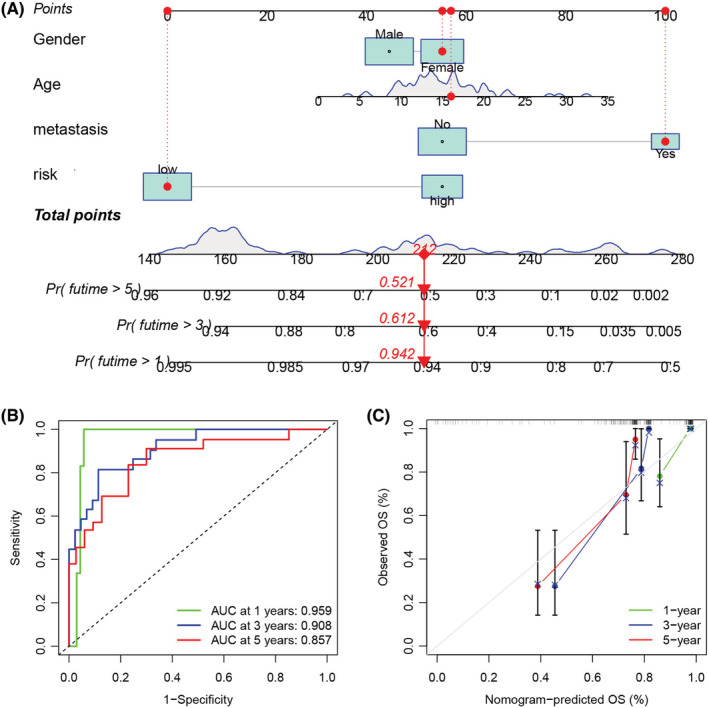
Construction of a nomogram after combining the risk scores and clinical properties to predict the survival of osteosarcoma patients. (A) A nomogram combined risk score and clinical information. (B) Time‐dependent receiver operating characteristic curves were used for validating the prognostic accuracy of the developed nomogram. (C) Calibration curves were developed for this nomogram, and they showed that the predicted and the actual 1‐, 3‐, and 5‐year overall survival values were in good agreement.

We developed a nomogram that used the risk score values of the patients as well as their available clinical data for accurately predicting the OS status of osteosarcoma patients (Figure 7A). The AUCs of ROC curves in the nomogram (Figure 7B) for the 1‐, 3‐, and 5‐year OS were 0.959, 0.908, and 0.857, respectively, demonstrating that this novel nomogram could accurately anticipate the prognosis of osteosarcoma patients. The calibration diagram (Figure 7C) showed that the predicted curve was very close to an ideal curve (a line drawn at an angle of 45° with slope  =  1 and passing through the origin of coordinate axes), which further indicated that the predicted and observed results were in good agreement.

### 3.5 Functional enrichment analysis

**FIGURE 8:**
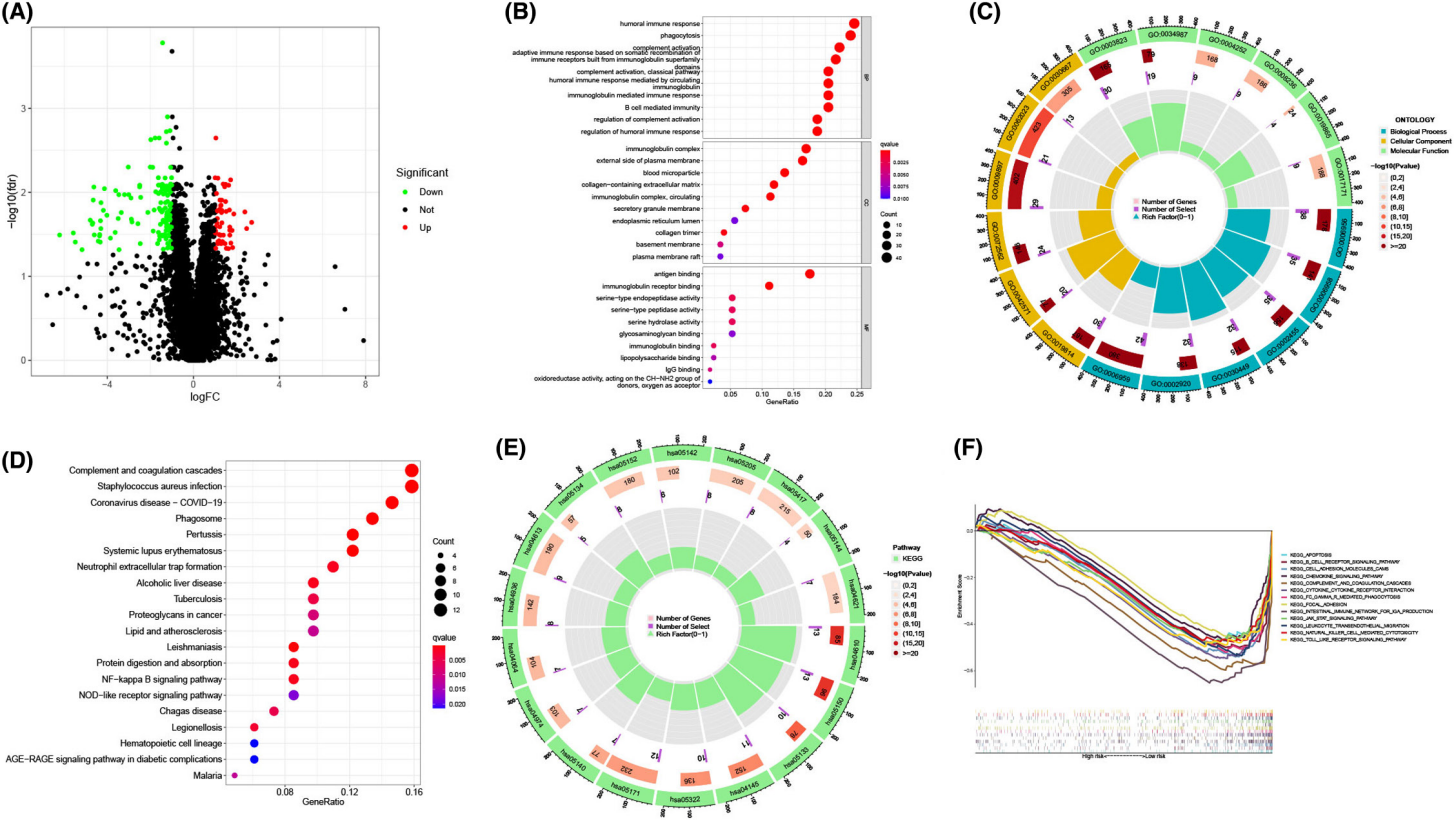
The functional enrichment analyses between the high‐risk and low‐risk patients. (A) Volcano plots for the differentially expressed genes (DEGs) were derived from the high‐ and low‐risk groups. The red dot indicated the upregulated genes, whereas the green dot denoted the downregulated genes. (B) Gene Ontology (GO) analysis for the DEGs between the high‐ and low‐risk groups. (C) Circle plots for the GO analysis. (D) KEGG analysis of the DEGs between the high‐ and low‐risk patients. (E) Circle plot of the KEGG analysis. (F) GSEA in the high‐ and low‐risk groups.

A total of 209 DEGs were identified between high‐ and low‐risk groups (Figure 8A). We carried out a GO analysis that indicated that the DEGs were enriched in the immune activation and immune response pathways. Figure 8B,C present the top 10 GO terms showing the highest significant enrichment. The KEGG analysis indicated that the DEGs were related to the inflammation‐ and immune‐related pathways, the top 20 KEGG terms were presented in Figure 8D,E. We also conducted the GSEA analysis for determining the potential biological processes and the pathways in the high‐ and low‐risk patients. The results indicated that the immune cell‐related pathways and immune signaling pathways were enriched in patients in the low‐risk group (Figure 8F), such as the chemokine signaling, B‐cell receptor signaling, and the NK‐cell‐mediated cytotoxicity pathways.

### 3.6 Tumor microenvironment and immune cells infiltrating

**FIGURE 9:**
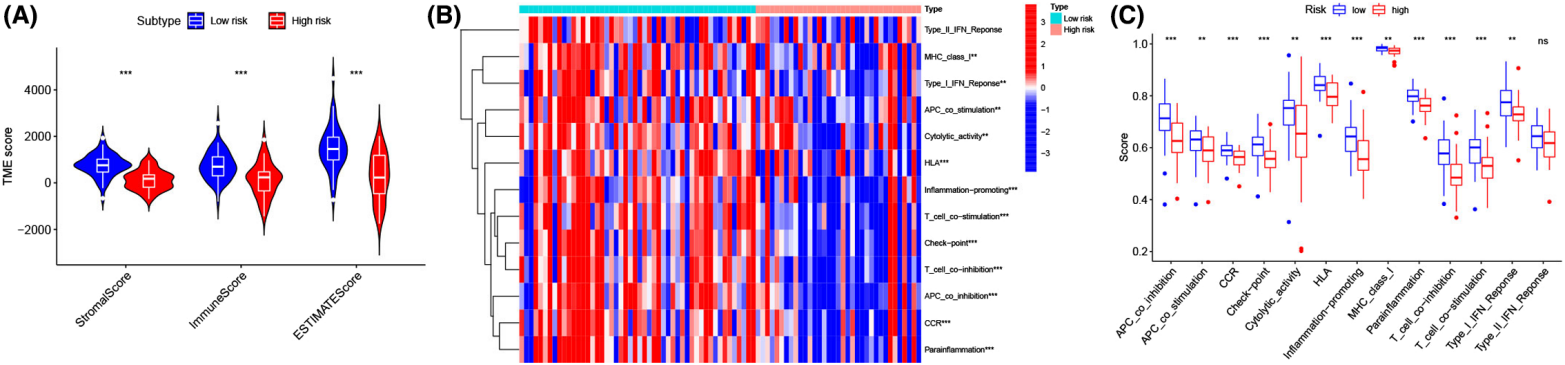
Correlation between the cuproptosis‐related lncRNA signature and the tumor microenvironment and immune functions. (A) Correlation analysis of ImmuneScore, StromalScore, and ESTIMATEScore with cuproptosis‐related lncRNA signature. The heatmap (B) and box plots (C) for the immune‐related functions noted in high‐ and low‐risk patients.

**FIGURE 10:**
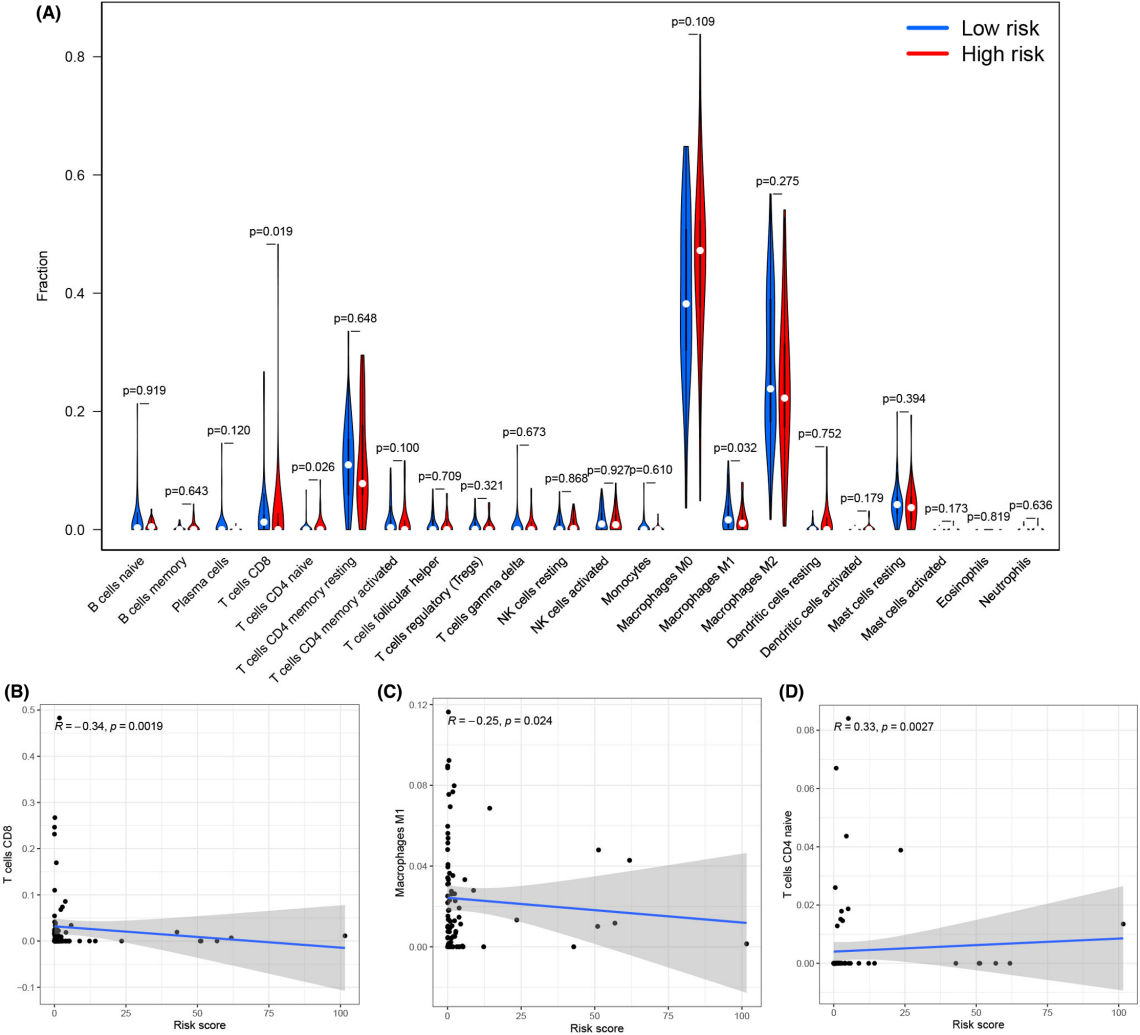
Relationship between the cuproptosis‐related lncRNA signature and immune cell infiltration. (A) Violin plots presented the different proportions of immune cell infiltration in the high‐risk and low‐risk patients. (B) CD8+ T cells were negatively associated with the risk scores of the patients. (C) M1 macrophages were negatively related to the risk scores of the osteosarcoma patients. (D) Naive CD4+ T cells were positively associated with the risk scores of the patients.

We assessed the number of immune and stromal components, independently, using the ESTIMATE method, and noted that the ImmuneScore, StromalScore, and ESTIMATEScore values were significantly higher in low‐risk patients than in high‐risk patients (Figure 9A). The above results implied that the low‐risk patients had more immune and stromal components. We carried out the ssGSEA analysis and noted that the 12 immune‐related functions (except Type‐II‐IFN‐Response) were significantly downregulated in the high‐risk patients (Figure 9B,C). We also employed the CIBERSORT technique for assessing the variations in the infiltration levels of the 22 immune cells in osteosarcoma patients. Violin plots (Figure 10A) revealed that high‐risk patients had a lower proportion of two immune effective cells (CD8+ T cells and M1 Macrophages) and a higher proportion of naive CD4+ T cells compared with the low‐risk patients. Furthermore, the Pearson correlation revealed that the risk scores of the patients were significantly and negatively related to the CD8+ T cell (Figure 10B, *R*  =  −0.34) and M1 macrophages (Figure 10C, *R*  =  −0.25), and significantly and positively related to the naive CD4+ T cells (Figure 10D, R  =  0.33).

### 3.7 Relationship between the risk scores and the ICGs

**FIGURE 11:**
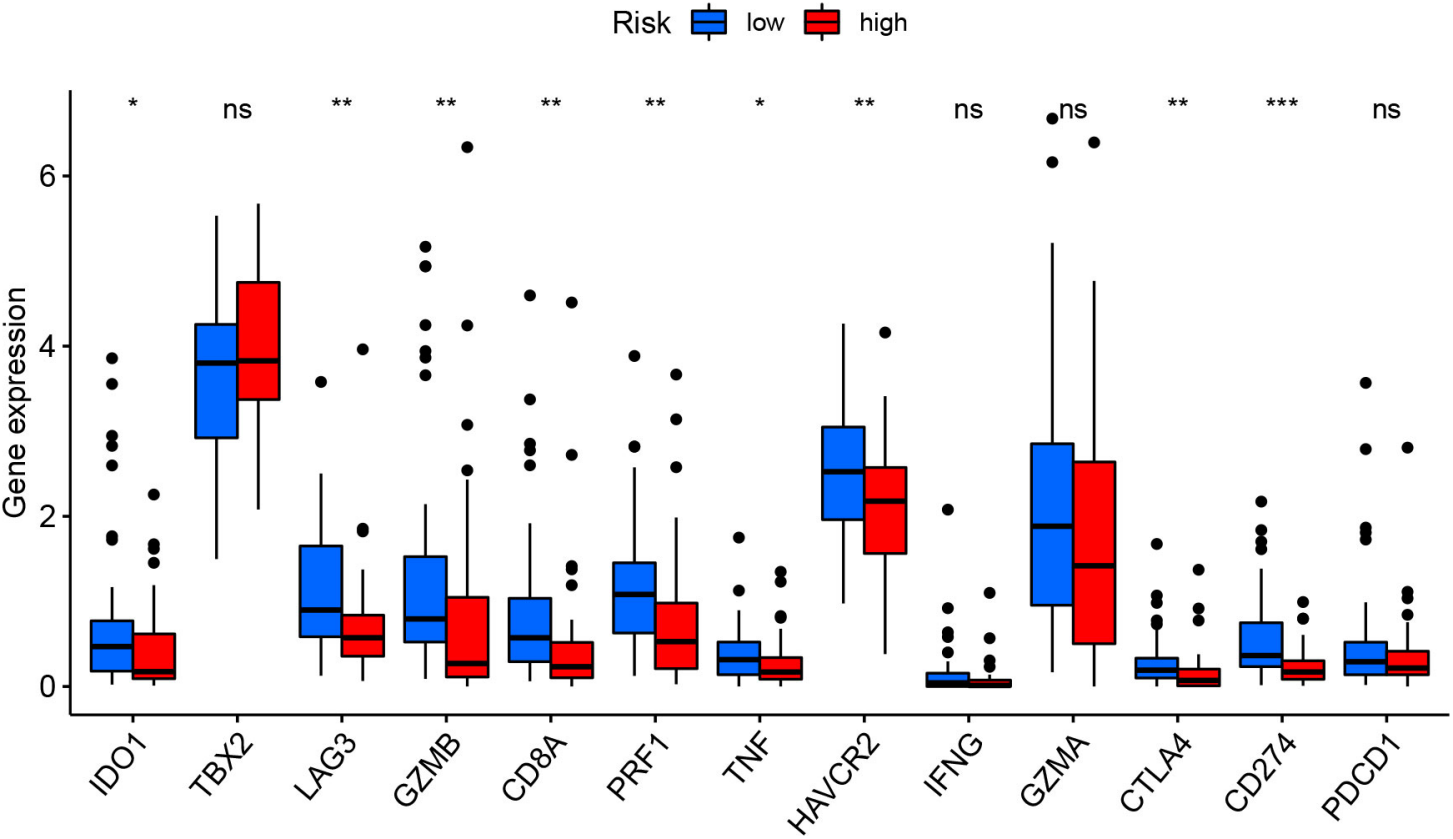
Different expressions of the immune check point‐related genes between the high‐risk and low‐risk patients. *p < 0.05, **p < 0.01, and ***p < 0.001.

The expression of the ICGs has been regarded as the biomarker for anticipating the effectiveness of immunotherapy in osteosarcoma patients. In this report, we noted that the low‐risk patients showed a higher expression level of the many ICGs (such as *IDO1*, *LAG3*, *GZMB*, *CD8A*, *PRF1*, *TNF*, *HAVCR2*, *CTLA4*, *CD274*, and *PDCD1*), indicating that these patients could respond better to immunotherapy (Figure 11).

## 4 DISCUSSION

The recent cancer statistics, published in 2022, by the American Cancer Society stated that despite advances in treatment techniques, osteosarcoma patients showed a 5‐year OS of only 68%.^2^ This highlights a crucial need to determine a specific biomarker for assessing the risk and monitoring the recurrence of osteosarcoma patients for precise treatment, especially for metastatic and recurrent patients. Recently, a novel cell death manner, cuproptosis, has been reported.^30^ Similar to the other cell death modalities, cuproptosis is also responsible for initiating the death of tumor cells^44^, ^45^ and also displays a high potential for application in tumor treatment.^35^ A lot of recent evidence has been presented that indicates that osteosarcoma onset and advancement are facilitated by the abnormal expression of the lncRNAs.^46^, ^47^ Additionally, abnormally expressed lncRNAs have been used as the predictive and prognostic biomarkers for osteosarcoma patients.^48^, ^49^ Furthermore, despite thorough investigation, we have not been able to conduct any previous studies that explored the correlation between the cuproptosis‐related lncRNAs and osteosarcoma.

In this study, we employed the TARGET database for screening the cuproptosis‐related prognostic lncRNAs using Pearson correlation analysis and univariate Cox regression analysis. In the training cohort, we developed a novel risk‐score model that was based on the four cuproptosis‐related lncRNAs (AL033384.2, AL031775.1, AC110995.1, and LINC00565), which were associated with tumorigenesis and prognosis. Amer et al. reported that LINC00565 was linked to the poor OS and progression‐free survival in glioblastoma multiforme.^50^ LINC00565 also targets the miR‐665/AKT3 axis in gastric cancer, enhances proliferation, and prevents apoptosis.^51^ Additionally, AL031775.1 was involved in several prognostic predicting models for patients with bladder cancer.^52^, ^53^, ^54^ In the current study, we showed that AL033384.2 was associated with a worse prognosis, and AL031775.1, AC110995.1, and LINC00565 were associated with a favorable prognosis. Subsequently, we classified patients into the high‐ and low‐risk groups depending on their median risk scores. According to the Kaplan–Meier survival curves, the high‐risk patients showed poor OS compared to the low‐risk patients. The AUC values of 1‐, 3‐, and 5‐year in ROC curves were >0.9, revealing that this model showed a strong prediction ability. Meanwhile, the AUCs were higher than that previous LncRNA signature,^37^, ^38^, ^39^ indicating a superior predictive ability for osteosarcoma patients. Furthermore, this model showed higher AUC values and C‐index, compared to factors like gender, age, and metastasis, implying that risk scores could accurately anticipate the prognosis of osteosarcoma patients compared to the traditional clinical risk markers. Importantly, these findings were confirmed in the validation and the entire cohorts. Consistently, the univariate and multivariate cox regression analyses confirmed that the risk scores could be considered a reliable and independent prognostic biomarker for osteosarcoma patients.

It is challenging to put a single indicator into clinical practice, even though the risk score shows the potential to be regarded as a promising predictor of clinical outcomes in osteosarcoma patients.^55^ Predictions made using the nomogram are more accurate and useful since they can take into account the relative importance of each variable.^56^, ^57^ Therefore, we integrated the risk score and other clinicopathological parameters to develop a nomogram. The results indicated that the AUC values of ROC curves were significantly higher compared to the single‐factor values (age, risk score, gender, and metastasis). The calibration diagram indicated that the predicted curve was closer to the ideal curve, proving that the developed nomogram could improve the predictive ability and accuracy to monitor osteosarcoma patients.

We also investigated the differential functions of the genes between the high‐ and low‐risk groups. The functional analyses of GO and KEGG implied that the DEGs in both groups were primarily enriched in the immune response and inflammatory response processes. Additionally, the GSEA and ssGSEA revealed that the low‐risk patients showed more prevalent immunological signaling and immune cell‐related pathways compared to the high‐risk patients. The aforementioned results suggested that low‐risk osteosarcoma patients may have a better immune activation capacity to trigger the anticancer responses. The hypothesis that low‐risk patients have a better prognosis is supported by the fact that an active immune response provides an environment for suppressing the development of cancer cells.

The tumor microenvironment (TME) includes the immune cells, stromal cells, cancer cells, and the extracellular matrix, and this complicated milieu played a vital role in promoting immune evasion, immune tolerance, tumorigenesis, and tumor progression.^58^, ^59^, ^60^ The balance of the TME is a critical factor that influences cancer patients' survival and response to immunotherapy.^61^ LncRNAs are involved in regulating the TME.^23^ Here, the ESTIMATE analysis revealed that the low‐risk patients showed higher ImmuneScore and StromalScore values, representing more immune or stromal cellular components in the TME of low‐risk patients. Consistent with this result, the immune cell infiltration analysis confirmed that the osteosarcoma patients categorized into the low‐risk group showed a high proportion of CD8+ T cells and M1 macrophages. The cytotoxic CD8+ cells exert a suppressive function and act as significant effector cells,^62^ which can interact directly with the tumor cells. Several studies indicated that the CD8+ T cells are positively related to the favorable clinical prognosis in numerous cancers.^40^, ^63^, ^64^, ^65^ Similarly, macrophages are known to be major components of the TME, including M1 macrophages and M2 macrophages, which usually maintain a balanced state.^66^ If M1 macrophages predominate, the balance may shift to an antitumor microenvironment.^67^, ^68^, ^69^ M1 macrophages can attack tumor cells and prevent tumor growth,^70^ and contribute to the favorable clinical outcome.^71^ Therefore, these two effective immune cell enrichments might partially explain the risk score of the model that could predict osteosarcoma patients' survival.

Recently, immune checkpoint‐modulating agents (represented by anti‐CTLA4 and anti‐PD antibodies), CAR‐T cells, and neoantigen vaccines have been seen to be successful in enhancing the antitumor effect in numerous types of cancer, bringing a paradigm shift to cancer treatment.^72^, ^73^, ^74^, ^75^ However, only a small number of osteosarcoma patients can benefit from immunotherapy, owing to the intratumor heterogeneity.^76^ Therefore, a biomarker is necessary for clinical management and prediction of immunotherapy efficacy in osteosarcoma. Previous studies showed that LncRNAs could target ICGs in cancer, which determines the immune activation level.^77^ In this study, we noted a higher expression level of the ICGs (*CTLA4*, *CD274*, *PDCD1*C, *HAVCR2*, and *LAG3*) in the osteosarcoma patients categorized into the low‐risk group, which suggested that these patients could gain more benefits from the immune checkpoint blockade therapy.

Nevertheless, this study also has a few shortcomings. First, the results presented in this study were solely confined to the data derived from the TARGET‐OS set, with a smaller sample size. Second, though we confirmed the effectiveness of this signature in the internal validation cohort, it is still necessary to verify the results using an external validation cohort with additional experiments. Third, the underlying mechanisms that differ in the high‐ and low‐risk patients were assessed using only the bioinformatics‐based prediction. We need to carry out additional in vitro and in vivo experiments for supporting these findings.

In conclusion, using the TARGET‐OS database, we initially categorized the osteosarcoma patients into the training and validation cohorts and then developed a risk score prognosis model using the cuproptosis‐related lncRNAs in the training cohort. We validated that this cuproptosis‐related lncRNA signature showed satisfactory performance and could accurately predict the OS for osteosarcoma patients, which was confirmed by the validation cohort and the entire cohort. The nomogram combining the risk score with the clinical characteristics could improve the prediction efficiency of the model. Furthermore, the low‐risk patients showed more immune active and more immune cells infiltrating, as well as had a better response to immunotherapy.

## AUTHOR CONTRIBUTIONS

**Shumin Ni:** Data curation (lead); writing – original draft (lead). **Jinjiong Hong:** Data curation (supporting); software (lead). **Weilong Li:** Formal analysis (equal); software (equal). **Meng Ye:** Conceptualization (equal); funding acquisition (equal); investigation (equal); visualization (equal); writing – review and editing (equal). **Jinyun Li:** Conceptualization (equal); formal analysis (equal); investigation (equal); supervision (equal); writing – review and editing (equal).

## FUNDING INFORMATION

The study was funded by the Zhejiang Key Laboratory of Pathophysiology (202204).

## CONFLICT OF INTEREST

The authors declare that there are no financial or other conflicts of interest associated with this study.

## ETHICAL APPROVAL STATEMENT

All data of this study were public and required no ethical approval.

## Supporting information


Table S1

## Data Availability

The data that support the findings of this study are publicly available from TARGET data sets (https://ocg.cancer.gov/programs/target).
